# Ovarian angiosarcoma: a case report and review of the literature

**DOI:** 10.1186/1752-1947-8-47

**Published:** 2014-02-12

**Authors:** Nausheen Yaqoob, Dalal Nemenqani, Hatem Khoja, Moemen Hafez, Asma Tulbah, Fuoad Al-Dayel

**Affiliations:** 1 King Abdul Aziz Specialist Hospital, As Salama Street, Taif, Kingdom of Saudi Arabia; 2 King Faisal Specialist Hospital and Research Centre, MBC-16, Riyadh 11211, Kingdom of Saudi Arabia

**Keywords:** Angiosarcoma, Ovary

## Abstract

**Introduction:**

Sarcomas of the ovary can either be histologically pure or can represent components of a more complex tumor. Ovarian angiosarcomas are rare, and probably arise from carcinosarcomas, teratomas or the rich ovarian vasculature. To date, only two small case series have been published, one with four cases and the other with seven.

**Case presentation:**

A 41-year-old Saudi woman presented to our gynecological clinic with abnormal vaginal bleeding. The initial clinical diagnosis was left ovarian cyst. The results of the remainder of her abdominopelvic examination were normal. Peri-operatively, the left ovarian mass resembled a hemorrhagic solid tumor. It was sent for frozen sectioning, which revealed it was an undifferentiated neoplasm. The final histopathological examination showed a vascular neoplasm showing vasoformative arborizing channels of variable sizes and shapes lined by atypical endothelial cells with intact capsule. Areas of necrosis were seen, along with fused anastomosing solid vascular area. She was diagnosed as having an angiosarcoma of intermediate grade, International Federation of Gynecology and Obstetrics stage IA.

**Conclusions:**

Patients with ovarian angiosarcomas most commonly present with abdominal pain, however some patients present with distant metastases, often in the lungs. Spread beyond the ovary is present at the time of diagnosis in most reported cases, with disease progression within less than a year after diagnosis. Cases of advanced stage disease behave aggressively and demonstrate poor response to surgery and chemotherapy, with an overall poor prognosis. They have a tendency for local recurrence and metastases, and prognosis is hence poor; the reported five-year survival rate is 10 percent to 35 percent, however, cases confined to the ovary have survived up to nine years.

## Introduction

Soft tissue sarcomas account for less than 1 percent of all malignancies. Gynecological sarcomas are rare and have poor prognosis. Among gynecological sarcomas, uterine sarcomas are the most common. Sarcomas of the ovary comprise less than 1 percent of ovarian malignancies, mostly seen as components of carcinosarcomas [1].

Angiosarcomas comprise only 2 percent of all soft tissue sarcomas [2]. They can occur in cutaneous, deep soft tissue, the breast, or visceral locations; cutaneous angiosarcoma in the head and neck region of older persons is the most common type. Angiosarcoma of deep soft tissue, except for the epithelioid variant, is very rare, and the prognosis almost always is poor. Visceral angiosarcoma have been reported in the liver, spleen, adrenal and thyroid glands, and heart, and less commonly in the vagina, vulva, cervix and rarely the ovary, with apparently fewer than 33 cases reported in the literature (although the validity of a considerable number of cases has been questioned).

Ovarian angiosarcomas are rare and probably arise from carcinosarcomas, teratomas or the rich ovarian vasculature. The development of a somatic malignancy, more frequently in the form of a sarcoma, is a well-known phenomenon in a germ cell tumor; however, the pathogenesis remains unclear. The sarcomatous element may appear in the primary tumor or manifest in recurrence or in the metastases. Dedifferentiation phenomenon, malignant transformation of certain mesenchymal elements within teratomas, origination from primitive germ cells and transformation of the blastematous stroma in yolk sac tumor are some of the proposed theories that have been put forward in the literature [2,3]. To date, only two small case series have been published, one with four cases and the other with seven [4,5]. Approximately 25 percent of ovarian angiosarcomas are associated with other tumors; five cases of angiosarcomas arising in mature cystic teratomas of the ovary have been described [4-8]. Of the seven cases of primary ovarian angiosarcoma reported by Neilson *et al*. [4], two arose in a dermoid cyst. Two of the cases of ovarian angiosarcoma were associated with a coexistent borderline or invasive epithelial neoplasm: one case with a mucinous cystadenocarcinoma and another with a serous neoplasm of low malignant potential [9,10]. Recently, cases of angiosarcoma arising in mucinous cystadenoma and ovarian fibroma have also been reported [11,12].

Ovarian angiosarcomas are usually unilateral and can occur at any age (range seven to 81 years, mean 48 years); with rare exceptions it is a disease found in premenopausal women and most cases are reported in women of childbearing age (<40 years). Only three cases have been reported in patients of postmenopausal age [12]. The most common presenting symptom is abdominal pain, however some patients present with distant metastases, often in the lungs. Other presenting features include disseminated intravascular coagulation (DIC) and combined ascites and pleural effusion. Most of the reported cases are unilateral, however, bilateral involvement of the ovaries has been recorded.

More than half of the cases were disseminated at the time of diagnosis and only a few of them were detected at stage I, with disease progression of less than a year. Cases of advanced stage disease behave aggressively and demonstrate poor response to surgery and chemotherapy, with an overall poor prognosis. Few cases have shown good response to a regimen of doxorubicin and ifosfamide.

## Case presentation

**Figure 1:**
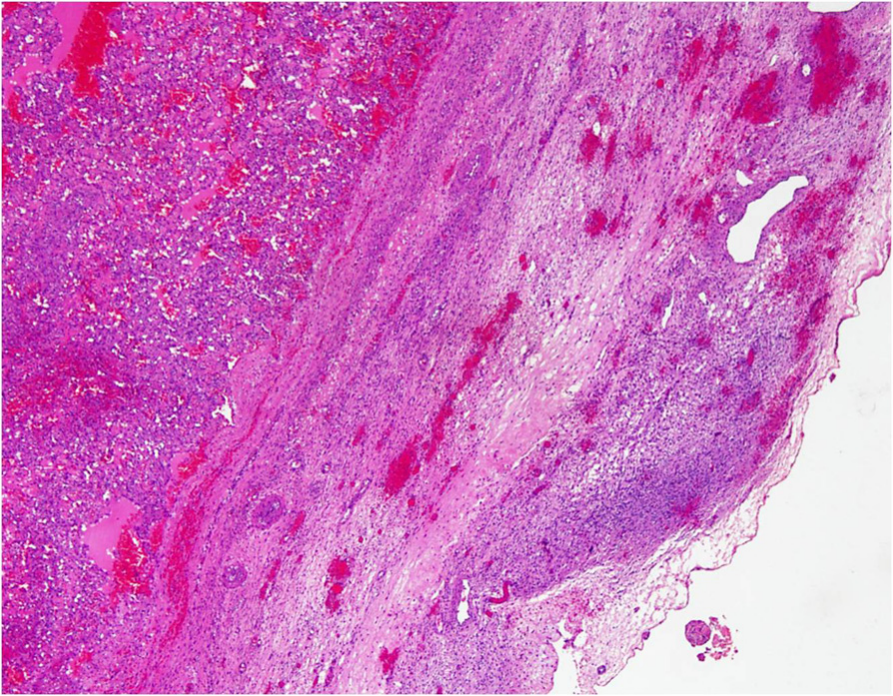
**Low power magnification showing ovarian parenchyma along with the vascular lesion.** Hematoxylin and eosin stain, 10×.

**Figure 2:**
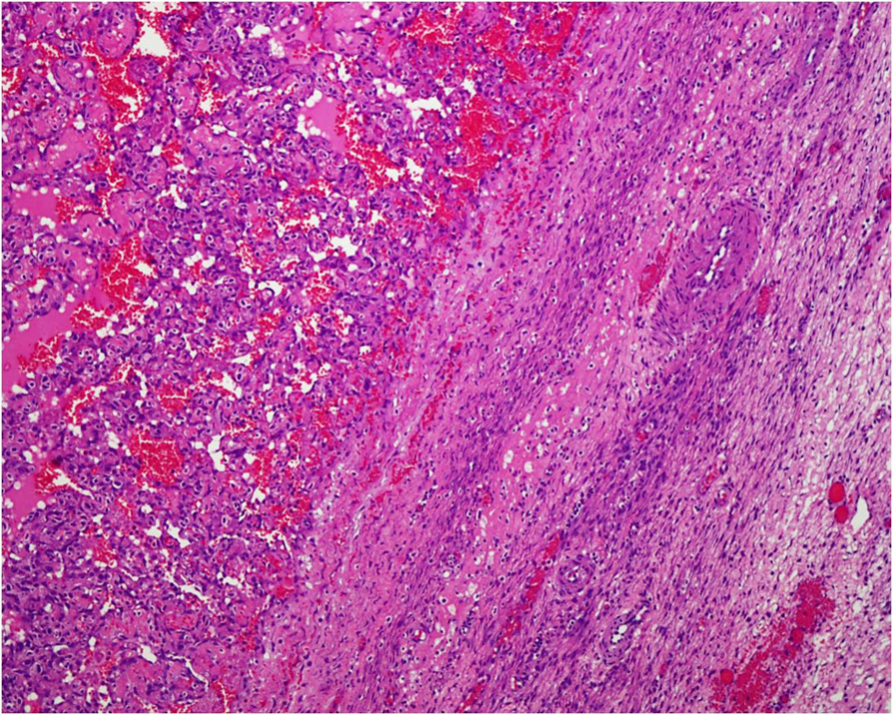
**Higher magnification of the tumor showing papillary vascular channels.** Hematoxylin and eosin stain, 10×.

**Figure 3:**
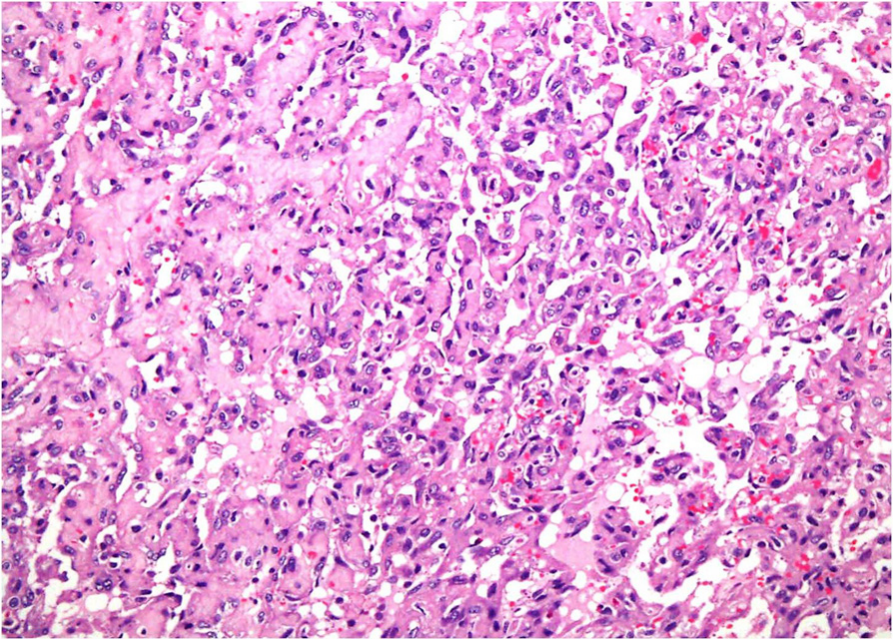
**Well-formed vasoformative channels.** Hematoxylin and eosin stain, 20×.

**Figure 4:**
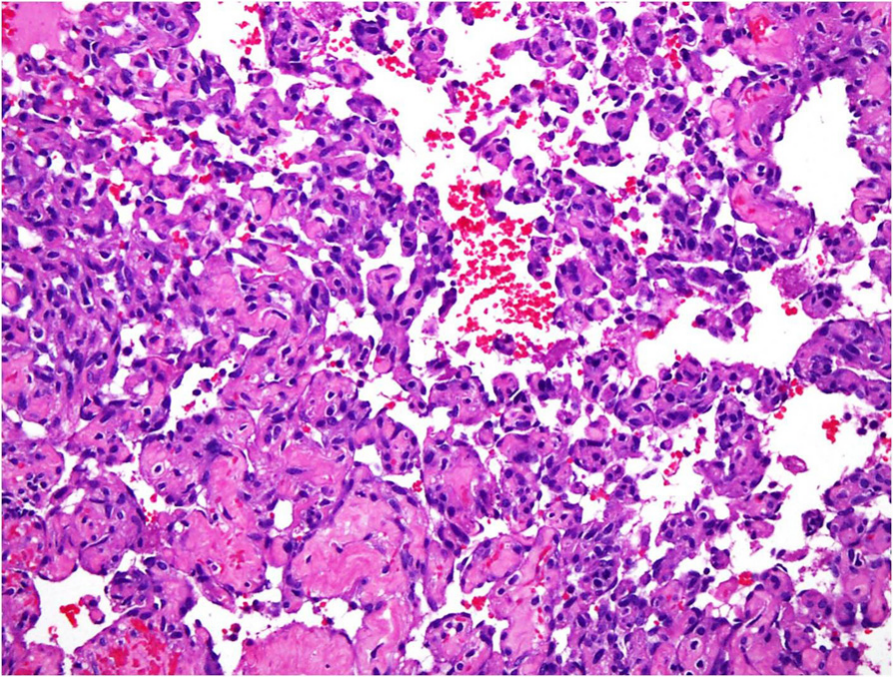
**High power magnification of well-formed vasoformative channels showing large pleomorphic nuclei.** Hematoxylin and eosin stain, 40×.

**Figure 5:**
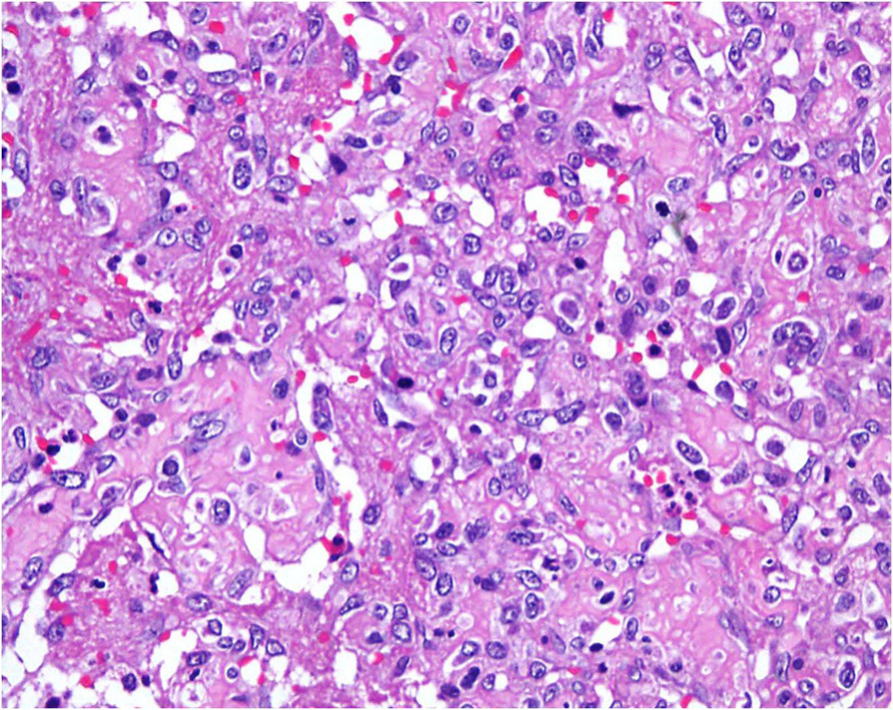
**Higher magnification showing solid areas with nuclear pleomorphism and scattered mitotic figures.** Hematoxylin and eosin stain, 40×.

**Figure 6:**
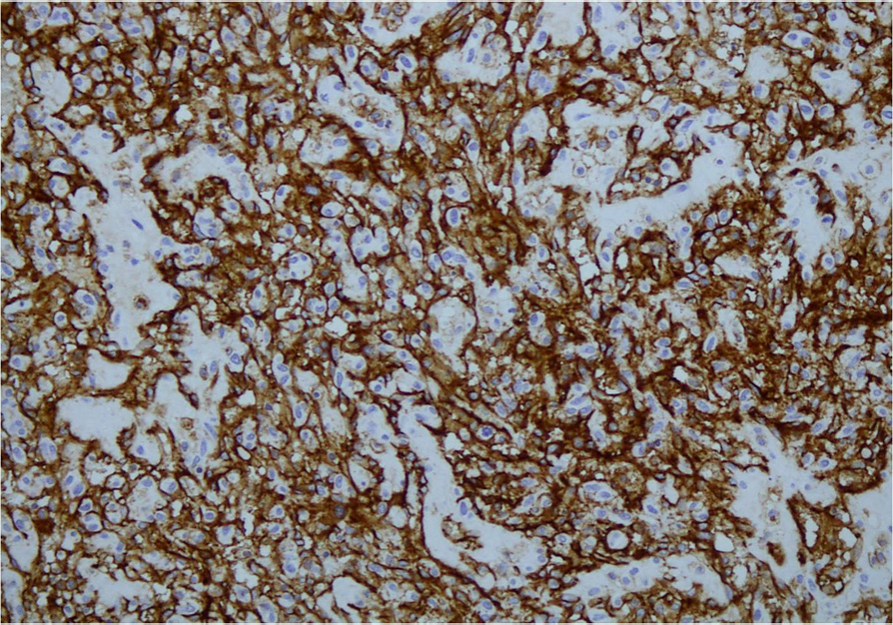
Tumor cells showing strong immunohistochemical positivity with CD 31.

A 41-year-old Saudi woman, para 7+0, presented to our gynecological clinic with abdominal pain and abnormal vaginal bleeding for the last six months. Her menstrual history was unremarkable. She had a history of a cesarean section three years previously and appendectomy performed a year ago. On examination, a palpable pelvi-abdominal mass was detected. Her hemoglobin was 10.1g and random blood glucose was 80mg. liver and renal function test results were within normal limits. An ultrasound study of her abdomen showed a normal-sized uterus displaced to the right side by a left ovarian mass, 8cm at its maximum dimension. No free fluid was seen. The initial clinical diagnosis was one of left ovarian cyst. The rest of her abdominopelvic sonographic examination was normal. Peri-operatively the mass resembled a hemorrhagic solid tumor. The left ovarian mass was sent for frozen sectioning, which revealed it was an undifferentiated neoplasm based on one frozen section; whether it was of benign or malignant nature could not be ascertained. The final specimen sent for histopathological examination consisted of a left salpingo-oophorectomy and incisional biopsy of the right ovary. Grossly, the left ovarian mass measured 7.0×6.0×2.0cm and was brownish and hemorrhagic, with solid and cystic areas. Permanent sections of the left ovarian mass showed a vascular neoplasm with adjacent compressed ovarian parenchyma at the periphery (Figures 1 and 2) showing vasoformative arborizing channels of variable sizes and shapes lined by atypical endothelial cells (Figures 3 and 4). The capsule was intact. Areas of necrosis were seen along with fused anastomosing solid vascular area (Figure 5). The mitotic count was four to five mitotic figures per 10 high power fields The results of special stains for glycogen and mucin were negative. No teratomatous element was identified. The tumor cells tested positive for CD31 (Figure 6), CD34, factor VIII and vimentin and negative for cytokeratin AE1/AE3 and low molecular weight cytokeratin. The proliferative index (Ki-67) was approximately 20 percent to 30 percent. Our patient was diagnosed as having an angiosarcoma, intermediate grade, International Federation of Gynecology and Obstetrics (FIGO) stage IA. A section of the right ovarian cyst wall showed a benign cyst with inflammation, extensive hemorrhage and fibroblastic proliferation. The results of a postoperative investigation for metastatic disease and evaluation for other possible primary sites were negative. Our patient was referred to our oncology center for further management, and subsequently lost to follow-up.

## Discussion

Sarcomas of the ovary either can be histologically pure or can represent components of a more complex tumor. Mature cystic teratoma (MCT) is the most common type of ovarian neoplasm, accounting for 26.5 percent to 44 percent of all primary ovarian tumors [13]. Malignant transformation in mature cystic teratoma is rare, seen in only 0.8 percent to 2 percent of cases, [8,14] and is mostly represented by squamous cell carcinomas (constituting about 80 percent of secondary somatic malignancies), followed by adenocarcinomas. Other benign and malignant somatic tumors include melanocytic nevi, skin adnexal tumors, carcinoids, carcinosarcoma, malignant melanoma, lymphoma, angiosarcoma, leiomyosarcoma, osteosarcoma, chondrosarcoma, fibrosarcoma, rhabdomyosarcoma, malignant fibrous histiocytoma and peripheral neuroectodermal tumor. The behavior of somatic malignancies in MCT is not determined by their derivation from germ cells but by their phenotype, and thus is similar to that of their sporadic counterpart.

The ovary is a common site for metastases and 5 percent to 10 percent of the ovarian malignancies are metastatic [15]. Details of spread of carcinomas to the ovary have been reported in the literature, however little information regarding the spread and frequency of metastatic sarcomas to the ovary has been described. Colon cancer is the most frequent primary malignancy of nongenital origin that metastasizes to the ovary, and infrequently gastrointestinal or urological sarcomas metastasize to the ovary. In 1990, Young and Scully described 21 cases of metastatic sarcoma relating to the ovaries [16]. The most common primary sites were the uterine corpus and cervix (11 of 21 cases). The most common extragenital primary tumor was leiomyosarcoma of the small intestine, followed by one case each of leiomyosarcoma of stomach, retrovesical leiomyosarcoma, fibrosarcoma of the anterior abdominal wall, presumed cardiac hemangiosarcoma, osteosarcoma of the maxilla, chondrosarcoma of the rib, and Ewing’s sarcoma of the pubic bone. Cases of primary angiosarcoma of the breast and subcutaneous tissue metastatic to the ovaries have been recorded [17,18]. In our patient’s case, no teratomatous element was found despite adequate sampling.

The presence of sarcoma cells in effusions is an exceedingly rare finding. Davidson and Abeler [19] reported the first case of a primary ovarian angiosarcoma with metastatic spread in the peritoneal cavity and para-aortic lymph nodes, presenting as malignant cells in two ascitic fluid specimens on immunocytochemistry. Platt *et al*. [6] also reported primary angiosarcoma of the ovary with large volume ascites, but cytological examination showed no malignant cells.

The microscopic appearance of angiosarcoma can vary, from focal or diffuse vasoformative components with dilated, slit-like, papillary or interconnecting vascular spaces to solid components that may be spindle sarcomatous or epithelioid. Microcystic/tubulocystic areas, and fascicular or reticular patterns can be seen. A diagnosis may be difficult if the vascular nature of the neoplasm is not evident and there are more poorly differentiated areas exhibiting solid growth patterns. The solid component can mimic fibrosarcoma, leiomyosarcoma or malignant fibrous histiocytoma. Immunohistochemically they are positive for endothelial markers including CD31 and CD34 (can be focally positive or negative) and factor VIII-related antigen. Focal positivity for smooth muscle actin, HHF35, pankeratin and epithelial membrane antigen (EMA) can be seen, however desmin is negative. The superiority of CD31 in terms of sensitivity and specificity for endothelial cells is well recognized, with reported sensitivity rates of 80 percent versus 62 percent in vasoformative and poorly vasoformative angiosarcomas, respectively. Angiosarcoma should be included in a panel in which an endothelial neoplasm is in the differential diagnosis. Factor VIII-related antigen is a marker of endothelial cell differentiation and its sensitivity decreases from 84 percent to 29 percent as the vasoformative areas become more solid and poorly differentiated [20].

The differential diagnosis includes hemangioma, epithelioid hemangiomas, infantile hemangioendothelioma and metastatic angiosarcoma, embryonal carcinoma, melanoma, poorly differentiated carcinoma and leiomyosarcoma. Pure primary sarcomas other than angiosarcoma that have been described to occur in the ovary include fibrosarcoma, rhabdomyosarcoma, leiomyosarcoma, chondrosarcoma, osteosarcoma, and malignant peripheral nerve sheath tumor. Benign vascular neoplasms of the ovary are uncommon. Case reports of isolated hemangiomas [21] of the ovary have been described, as well as those associated with generalized hemangiomatosis. Subtypes of hemangioma have been reported in the ovary, including a mitotically active epithelioid vascular tumor that behaved in a benign fashion [22]. Baker, Rosai, and Yough [23] reported florid benign vascular proliferation in ovarian teratomas (MCT and immature teratoma) that may be confused with a vascular neoplasm probably induced by growth factors liberated by extensive neural component seen in these tumors. In general, hemangiomas of the ovary are located in the medulla and are of the cavernous type. Distinction from a malignant mixed Müllerian tumor is based on the presence of a malignant epithelial component, as well as its occurrence in an older population. Poorly differentiated, more solid and spindle-shaped angiosarcomas may also mimic sarcomatoid carcinomas on histological evaluation. If close to an epithelioid malignancy, epithelioid angiosarcoma may easily be mistaken for a poorly differentiated carcinoma. The presence of histologically better differentiated carcinomatous areas, as well as positive staining for keratin (albeit sometimes only focally) in the sarcomatoid areas may be helpful. Conversely, immunopositivity for CD31 or von Willebrand factor would rule out carcinoma. Cases of angiosarcoma with a more fascicular growth pattern in poorly differentiated areas may closely mimic other mesenchymal tumors, such as a leiomyosarcoma. The presence of areas that are suggestive of vascular differentiation may be a helpful clue; however, distinction relies ultimately on the focal presence of more typical morphologic features and the immunohistochemical results. Because of the rarity and morphologic heterogeneity of ovarian angiosarcoma, immunopositivity with a specific endothelial marker (CD31 or von Willebrand factor) is a diagnostic prerequisite.

The behavior of somatic malignancies in MCT is not determined by their derivation from germ cells but by their phenotype, and is similar to that of their sporadic counterparts; therefore, treatment should target the somatic malignancy rather than the germ cell tumor.

Such malignancies respond poorly to adjuvant chemotherapy. The most common chemotherapeutic regimen used to treat is a combination of doxorubicin and/or ifosfamide. The combination of mesna, doxorubicin, ifosfamide and dacarbazine (MAID) chemotherapy has also been reported, however the efficacy is still uncertain. More than 60 percent of all reported cases of ovarian angiosarcoma were detected at stages III and IV. They have a tendency for local recurrence and metastases, and prognosis is poor; the reported five-year survival rate is 10 percent to 35 percent [24]. The survival for all cases ranged from 18 days to nine years, however, no stage III or IV patient with a reported follow-up has survived more than 30 months. The only exception is in a patient described by Platt *et al*. [6], who describes complete resolution of a stage IV angiosarcoma after five cycles of MAID chemotherapy. There are seven documented cases of patients with stage I disease surviving up to nine years, with one even becoming pregnant [2,4,5,25].

## Conclusions

Examination of multiple sections of an ovarian neoplasm, use of immunohistochemical studies in cases of poorly differentiated tumors and awareness of occurrence of angiosarcoma in ovary may result in more cases being identified and reported.

## Consent

Written informed consent was obtained from the patient’s next of kin for publication of this case report and any accompanying images. A copy of the written consent is available for review by the Editor-in-Chief of this journal.

## Competing interests

The authors declare that they have no competing interests.

## Authors’ contributions

All authors have contributed in the writing of the manuscript and all authors read and approved the final manuscript.
